# The relationship between inflammation and new bone formation in patients with ankylosing spondylitis

**DOI:** 10.1186/ar2496

**Published:** 2008-09-01

**Authors:** Xenofon Baraliakos, Joachim Listing, Martin Rudwaleit, Joachim Sieper, Juergen Braun

**Affiliations:** 1 Rheumazentrum Ruhrgebiet Herne, Ruhr-University Bochum, Landgrafenstr. 15, 44652 Herne, Germany; 2 German Rheumatism Research Center, Charitéplatz 1, 10117 Berlin, Germany; 3 Rheumatology, Charité, Campus Benjamin Franklin, Hindenburgdamm 30, 12200 Berlin, Germany

## Abstract

**Introduction:**

Spinal inflammation as detected by magnetic resonance imaging and new bone formation as identified by conventional radiographs are characteristic of ankylosing spondylitis. Whether and how spondylitis and syndesmophyte formation are linked are unclear. Our objective was to investigate whether and how spinal inflammation are associated with new bone formation in ankylosing spondylitis.

**Methods:**

Spinal magnetic resonance images and conventional radiographs from 39 ankylosing spondylitis patients treated with anti-tumour necrosis factor (anti-TNF) agents at baseline and after 2 years were analysed for syndesmophyte formation at vertebral edges with or without inflammatory lesions at baseline.

**Results:**

Overall, 922 vertebral edges at the cervical and lumbar spine were analysed. At baseline, the proportion of vertebral edges with and without inflammation (magnetic resonance imaging) that showed structural changes (conventional radiographs) was similar (in total, 16.6% of all vertebral edges in 71.4% of patients). From the perspective of syndesmophyte formation (n = 26, 2.9%) after 2 years, there were more vertebral edges without (62%) than with (38%) inflammation at baseline (*P *= 0.03). From the perspective of spinal inflammation at baseline (n = 153 vertebral edges), more syndesmophytes developed at vertebral edges with (6.5%) than without (2.1%) inflammation (*P *= 0.002, odds ratio 3.3, 95% confidence interval 1.5 to 7.4). Inflammation persisted in 31% of the initially inflamed vertebral edges (n = 132), and new lesions developed in 8% of the vertebral edges without inflammation at baseline (n = 410). From the perspective of spinal inflammation after 2 years (n = 72 vertebral edges), 5.6% of the vertebral edges showed syndesmophyte development in contrast to 1.9% of the vertebral edges with new syndesmophytes without inflammation (*P *= 0.06).

**Conclusions:**

These findings obtained in patients treated with anti-TNF agents suggest linkage and some dissociation of inflammation and new bone formation in ankylosing spondylitis. Although syndesmophytes were also found to develop at sites where no inflammation had been seen by magnetic resonance imaging at baseline, it was more likely that syndesmophytes developed in inflamed vertebral edges. More effective suppression of spinal inflammation may be required to inhibit structural damage in ankylosing spondylitis.

## Introduction

Ankylosing spondylitis (AS) is a frequent chronic inflammatory rheumatic disease that already affects the axial skeleton at a young age [1], starting in the sacroiliac joints and later spreading to the spine [2]. Active inflammatory spinal lesions as detected by magnetic resonance imaging (MRI) [3] and chronic structural changes such as syndesmophytes as demonstrated by conventional radiography [4] are characteristic of AS and contribute to both decreased spinal mobility and functional impairments of these affected patients [5]. Conventional spinal x-rays are still the gold standard for assessment of structural changes in AS [6,7], whereas MRI techniques using either short-tau-inversion-recovery (STIR) sequences [2,8] or T1-post-gadolinium (T1-post-Gd) [9] are best for assessing spinal inflammation.

For quantification of structural spinal changes in conventional radiographs, the modified Stokes AS spinal score (mSASSS) [10] is the best currently available scoring method based on the OMERACT (Outcome Measures in Rheumatology) filter [11]. For a sufficient sensitivity to change in depiction of structural spinal changes in AS when using conventional radiographs, a minimal observation period of 2 years is required [12]. Similarly, for assessment and quantification of inflammatory spinal changes, the AS-spinal-MRI scoring system [13] has shown good discriminatory capacity, validity, and sensitivity to change in MRI examinations for periods of between 6 weeks [14] and 2 years [15-17].

Tumour necrosis factor-alpha (TNF-α) plays a key proinflammatory role in AS [18,19] given that spinal inflammation was shown to be associated with the presence of TNF-α mRNA [18] and protein [20] in affected joint and bone structures. Accordingly, inhibition of TNF-α was found to substantially improve signs and symptoms of AS patients [21-23]. Similarly, using MRI, a significant decrease of inflammatory lesions already after 6 weeks of therapy [14] and ongoing improvement of spinal inflammation for up to 2 years [15,16] of continuous treatment have been reported. However, some inflammatory lesions were still present even after this period [15,17,24].

Chronic changes in the thoracic spine cannot be reliably assessed by conventional x-rays but a valid quantification of such lesions is possible in the cervical and the lumbar spine [4]. Since MRI is able to visualise the entire spine, it is now clear that the lower part of the thoracic spine is most frequently involved in AS [3,17,25]. This is one possible reason why so far it has not been possible to demonstrate major inhibition of structural damage in AS patients on anti-TNF therapy [26-28]. Nevertheless, a direct link between spinal inflammation and future radiographic progression has not been sufficiently proven until now. Data from animal models have even suggested that inflammation and new bone formation are uncoupled [29,30]. In this study, we examined the relationship of MRI-proven spinal inflammation at baseline (BL) with respect to structural deterioration depicted by conventional radiographs after 2 years in AS patients treated with anti-TNF-α agents.

## Materials and methods

Overall, conventional radiographs of 39 AS patients who were diagnosed according to the modified New York criteria for diagnosis of AS [31] were analysed. All patients had participated in clinical studies on anti-TNF therapy with infliximab (n = 26) [21,32] or etanercept (n = 13) [24] for at least 2 years. All patients whose images were analysed had already signed informed consent forms for the radiographic images to be taken and analysed, according to the ethics committees of the participating centres which approved the original studies. None of these patients received antiresorptive bone therapy such as bisphosphonates or other drugs. Inclusion criteria were the availability of complete sets of MRIs with STIR and T1-post-Gd sequences and of conventional radiographs (see below) at the time point of presentation (BL) and after 2 years of follow-up (2yFU). All MRI and x-ray examinations were conducted using the same standardised protocol, as recently reported [4,13,24].

### Quantification of inflammatory and chronic spinal lesions

Depiction and quantification of inflammatory and chronic spinal lesions were performed on the basis of vertebral edges (VEs) in this study, in accordance with recent results [4,33,34]. This method was the most specific and also the most sensitive to change for the depiction of structural spinal changes in patients with AS as compared with assessments on the patient level or on the basis of change scores. For the assessment of structural changes by conventional x-rays, complete sets of radiographs of the cervical and the lumbar spine in the lateral view at BL and 2yFU were taken. Because of the known technical problems in the assessment of the thoracic spine in standard x-rays [4], this part of the spine was not available for analysis. As recently proposed, we defined 'definite radiographic damage' as the appearance of at least one syndesmophyte in each individual VE since this was the most reliable parameter to depict disease-related damage or change between follow-ups in patients with AS [4]. Similarly, to assess change over time, 'definite radiographic progression' was defined as the development of new syndesmophytes or ankylosis in individual VEs [4]. In comparison, for the assessment of inflammatory changes by MRI, only the cervical and the lumbar spinal segments were analysed for spinal inflammation, similar to the available x-rays. To be even more precise in the relationship of inflammatory activity in MRI and new bone formation of the same VEs in conventional radiographs, a VE in MRI was defined as 'positive' for inflammation if the inflammatory activity was present in the anterior half of the VE only. For analysis of the relationship between spinal inflammation at BL and radiographic progression after 2 years, all individual VEs were examined by MRI for signs of inflammation at BL and the same VEs were compared for development of new syndesmophytes in conventional radiographs at BL and 2yFU.

### Statistical analysis

The Fisher exact test was used for comparison between different subgroups such as those with VEs and without spinal inflammation or with and without definite radiographic damage and progression. Furthermore, subgroups of VEs with signs of inflammation as well as radiographic progression were selected based on the definition of radiographic progression as defined by four different subsamples. In each of those subsamples, two conditional probabilities were compared: first, the likelihood of radiographic progression in VEs with signs of inflammation and, second, the likelihood of radiographic progression in VEs without inflammation. The Wilcoxon test was applied to compare both paired proportions across all VEs of the subsamples.

## Results

### Analysis of baseline data

Overall, 922 VEs of the cervical spine and lumbar spine of 39 AS patients were available for analysis at BL and at 2yFU. Missing data are explained by incomplete radiographic image sets since, for technical reasons, not all VEs could be captured on some films [4]. Spinal inflammation at BL (STIR MRI sequence) was present in 153/922 (16.6%) VEs, whereas no signs of inflammation were seen in 769/922 (83.4%) VEs. At least one vertebral body with signs of inflammation was found in 28/39 (71.8%) patients. The BL data as assessed by T1-post-Gd MRI showed similar results (Table 1). At BL, the VEs with or without spinal inflammation in MRI showed similar proportions of definite radiographic damage at BL (17.6% versus 15.6%, respectively; *P *> 0.05). Thus, there was no difference at BL in the proportion of VEs showing structural changes (syndesmophytes) in these subgroups.

**Table 1:** Baseline data on inflammation and occurrence of definite radiographic damage as assessed by both magnetic resonance imaging sequences

MRI sequence	Inflammation/radiographic damage	Proportion (number) of vertebral edges at baseline	*P *value
STIR	Any inflammatory lesions	16.6% (153/922)	-
	No inflammatory lesions	83.4% (769/922)	
	Definite radiographic damage with inflammation	17.6% (27/153)	0.53
	Definite radiographic damage without inflammation	15.6% (120/769)	
T1-post-Gd	Any inflammatory lesions	10.3% (95/922)	-
	No inflammatory lesions	89.7% (827/922)	
	Definite radiographic damage with inflammation	21.1% (20/95)	0.13
	Definite radiographic damage without inflammation	15.1% (125/827)	

There was no difference in the proportion of inflammatory lesions and occurrence or absence of syndesmophytes at baseline. MRI, magnetic resonance imaging; STIR, short-tau-inversion-recovery; T1-post-Gd, T1-post-gadolinium.

### Analysis of the 2-year follow-up data

**Figure 1:**
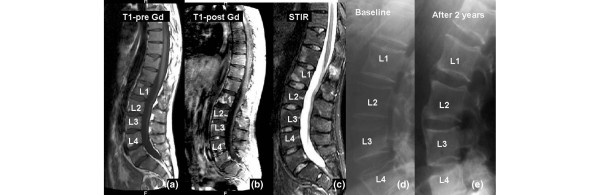
Formation of new syndesmophytes in the upper and lower edges of L1/L2 and L2/L3. **(a) **T1-pre-gadolinium (T1-pre-Gd) image. Spinal inflammation in the same area is assessed by both magnetic resonance imaging (MRI) sequences: **(b) **T1-post-gadolinium (T1-post-Gd) and **(c) **short-tau-inversion-recovery (STIR). Inflammation at baseline is seen as a 'spot' in the T1-post-Gd image only after application of gadolinium. The STIR image shows signs of inflammatory activity in the same vertebral regions. Formation of new syndesmophytes in the upper and lower edges of L1/L2 and L2/L3 is seen in conventional x-rays developing from **(d) **baseline to **(e) **2 years later.

Radiographic progression based on the development of new syndesmophytes was seen in 26/922 VEs (2.8%) after 2 years. Of those, 10 VEs (38%) had initially shown signs of inflammation as detected by MRI whereas the remaining 16 VEs (62%) had no such changes at BL (*P *= 0.006 between VEs with and without BL inflammation) (Table 2). The analysis based on the T1-post-Gd MRI sequences revealed similar results (data not shown). In the prospective data analysis, definite radiographic progression was found significantly more often in VEs with MRI-proven inflammation at BL (10/153 VEs, 6.5%, 95% confidence interval [CI] 3.6% to 11.6%) than in VEs without BL inflammation in MRI (16/769 VEs, 2.1%, 95% CI 1.3% to 3.4%) (*P *= 0.006, odds ratio [OR] 3.3, 95% CI 1.5 to 7.4). This was similar for the T1-post-Gd MRI sequences (Table 2 and Figure 1).

**Table 2:** Proportion of vertebral edges showing development of new syndesmophytes at 2-year follow-up according to baseline status of inflammation as assessed by both magnetic resonance imaging sequences

MRI sequence	Inflammation status	Proportion (number) of vertebral edges with development of new syndesmophytes after 2 years	
STIR	Inflammation at baseline	6.5% (10/153)	*P *= 0.006, OR 3.3, 95% CI 1.5 to 7.4
	No inflammation at baseline	2.1% (16/769)	
T1-post-Gd	Inflammation at baseline	6.3% (6/95)	*P *= 0.043, OR 2.7, 95% CI 1.1 to 7.0
	No inflammation at baseline	2.4% (20/827)	

CI, confidence interval; MRI, magnetic resonance imaging; OR, odds ratio; STIR, short-tau-inversion-recovery; T1-post-Gd, T1-post-gadolinium.

### Relation of radiographic progression to persistent spinal inflammation after 2 years

Follow-up MRIs after 2 years were available in 23/39 patients (59%). In those, 542 VEs could be scored. There were 132 VEs with inflammation (STIR sequence) at BL (25%) and 410 VEs without (75%). After 2 years, there were 72 VEs with inflammation (13%) and 410 VEs without (87%). In more detail, there were 41/132 VEs (31%) with and 91/132 VEs (69%) without persistent inflammation at follow-up, while 31/410 VEs (8%) without BL inflammation showed (new) inflammatory lesions and 379/410 VEs (92%) remained without such changes. Thus, after 2 years, there were still 72/542 of the VEs (13.3%) showing inflammation. Development of syndesmophytes was found in 2/41 VEs (4.9%) and in 2/31 VEs (6.5%) that showed inflammatory lesions at 2yFU. In contrast, syndesmophytes were also developed in 4/91 VEs (4.4%) and in 5/379 VEs (1.3%) that did not show inflammatory lesions at 2yFU. Thus, 4/72 VEs (5.6%) developed syndesmophytes on the basis of inflammation at 2yFU in contrast to 9/470 VEs (1.9%) that developed syndesmophytes not based on inflammation at 2yFU (*P *= 0.06). The T1-post-Gd MRI data showed similar results (data not shown).

## Discussion

The results of the present study suggest that spinal inflammation and new bone formation are both uncoupled and linked in AS since (a) the majority of syndesmophytes developed without MRI evidence of spinal inflammation at BL and (b) the proportion of VEs that developed syndesmophytes within 2 years was threefold higher when spinal inflammation was present at BL (compared with edges without BL inflammation). Since this was observed in patients under anti-TNF-α treatment, these data can be interpreted only on this basis. It will be important to study whether this is also true for patients just treated with nonsteroidal anti-inflammatory drugs or other agents. Nevertheless, the data may indicate that spinal inflammation was not sufficiently suppressed by TNF blockers in this 2-year time period.

In this study, the primary outcome was based on the analysis of VEs because the patterns of spinal inflammation and the development of syndesmophytes are likely to differ in and between individuals. It was no surprise, therefore, that when we did the analyses on an individual patient basis, no differences in the proportions of patients with and without spinal inflammation with respect to the development of future syndesmophytes were found. This may also be explained by the relatively small number of patients in this cohort. However, since there clearly were patients who developed syndesmophytes irrespective of BL inflammation, we do believe that it is more useful to do the calculations on the basis of VEs rather than on the patient level.

VEs that showed persistent inflammation seemed to be more prone to develop new syndesmophytes after 2 years as compared with those edges where inflammatory lesions disappeared after anti-TNF treatment. Indeed, in this study and in others, it has been shown that spondylitis may still be present after 2 years of anti-TNF therapy – even in patients with definite clinical improvement [15,24]. In addition, the analysis of only the edges that were inflammation-free at 2yFU showed that the tendency for the development of new syndesmophytes was stronger for those edges that showed inflammatory lesions at BL as compared with those edges that had not been inflamed in either the BL or the 2yFU. Nevertheless, our findings are in line with previous data of ours [26,28] and of other groups [35,27] showing that radiographic progression in AS is not or not completely inhibited by TNF blockers.

Since the key feature of AS, much unlike rheumatoid arthritis [36], is new bone formation rather than osteodestruction, there is reason to consider different mechanisms for structural change which appear on radiographs in these diseases. In AS, uncoupling of spinal inflammation and new bone formation has recently been suggested [37]. The data of our study show that about 60% of all syndesmophytes that developed did not show inflammation as detected by MRI. Since the sensitivity of MRI to demonstrate spinal inflammation in AS is not precisely known [9], the question of whether it was possible to really detect all cases of spondylitis has to remain open and should be studied further. Furthermore, it cannot be excluded that inflammation has occurred at some point before and/or during the study. In this study, new spondylitis lesions developed in 8% of the VEs investigated. Recent immunohistological data showed low-grade spinal inflammation in biopsy specimens obtained at spinal surgery of AS patients who had undergone MRI before surgery, and no active inflammatory lesions had been detected by appropriate MRI sequences [38]. Thus, since we did not perform MRIs in between, we do not know whether or for how long spinal inflammation may have occurred in the patients included in this study.

This is the first study based on patient data on this issue – even though we cannot exclude that the treatment of the patients had an impact on the results. Indeed, there is some evidence that blocking TNF-α may reverse the physiologic inhibition of osteoblast function and stimulate osteoclast resorption [39]. TNF and other proinflammatory cytokines are known to promote bone formation by upregulating the expression of Dickkopf-1, a key target gene of TNF and an inhibitor of osteophyte regulators [29]. Thus, by inhibiting TNF and Dickkopf-1, TNF blockers may even block negative influences on syndesmophyte formation after sufficient suppression of inflammation [29]. The hypothesis that new bone formation in AS is uncoupled from inflammation has been supported by animal models showing that TNF inhibition did not affect joint ankylosis [30].

Recent biomarker data generated from ASSERT (Ankylosing Spondylitis Study for the Evaluation of Recombinant Infliximab Therapy) [22,40] showed that previously low levels of osteocalcin and bone alkaline phosphatase were significantly increased under infliximab therapy [41]. Furthermore, anti-TNF therapy was shown to decrease osteoclast precursor cells [42] and to increase bone mineral density [43] in AS patients. Thus, there is evidence from patient-derived data that anti-TNF agents increase bone mass. On the other hand, clinical experience may suggest that syndesmophytes grow especially at locations where spondylitic lesions had occurred. One example is the radiologic appearance of spondylitis anterior, the well-known shiny corners or Romanus lesions [44]. Furthermore, it was already described some decades ago in histological studies that inflammatory spinal lesions precede new bone formation in AS patients [45]. Our study shows that the likelihood that syndesmophytes developed was much higher for VEs with MRI evidence of inflammation than for those without (OR > 3). This suggests that there is some link between inflammation and new bone formation, even though that may not be a mandatory prerequisite for syndesmophyte development. Furthermore, as indicated by the analysis of T1-post-Gd sequences, which are more specific but not as sensitive as STIR in the depiction of spinal inflammation in AS [9], formation of new syndesmophytes occurred in VEs with persistent inflammation after 2 years (4.3%), whereas this was not the case in edges without persistent inflammation.

The nature and the length of the time interval between inflammation and new bone formation are unclear. Animal models imply that new bone formation in AS is mainly due to 'response to an inflammation-based bone-resorptive phase which serves as a stress factor' and is followed by enchondral new bone formation leading to bony bridges and vertebral fusion [37]. Although it is unclear whether and how such findings are relevant for human disease, it is conceivable that there may be a disease stage at which new bone formation occurs without much actual inflammation; this, however, remains to be shown. Thus, it seems possible that both hypotheses are true; this implies that inflammation and new bone formation in AS are not completely uncoupled in AS, as recently proposed [37], but are at least partially linked.

While osteodestructive lesions in rheumatoid arthritis can already be inhibited by anti-TNF-α therapy after 1 year [36], inhibition of the osteoproliferation in patients with AS may need longer treatment [28]. However, since it was shown that the spinal inflammation is not completely inhibited by anti-TNF therapy in this and other studies after 2 years [35,27], there are also other factors [4] to be considered to explain this major difference to response to therapy between these two diseases. This includes the fact that only historical cohorts are currently available for comparison in relevant studies [35,27].

In summary, in patients treated with anti-TNF-α, new bone formation occurred almost threefold more often in regions with MRI-proven spinal inflammation at BL, and, in the same cohort, most of the newly developed syndesmophytes occurred in VEs without evidence of inflammation at BL. These findings suggest both a link and some dissociation of inflammation and radiographic damage. There is no evidence for a major uncoupling of these characteristic features in AS. Thus, it seems still possible that more effective suppression of spinal inflammation may lead to a stronger inhibition of structural damage in AS.

## Conclusion

In patients treated with anti-TNF-α, new bone formation seems to occur almost threefold more often in regions with MRI-proven spinal inflammation at BL. But, similarly, some of the newly developed syndesmophytes may also occur in VEs without evidence of inflammation at BL. These findings suggest both a link and some dissociation of inflammation and radiographic damage. There is no evidence for a major uncoupling of these characteristic features in AS. It seems still possible that more effective suppression of spinal inflammation may lead to a stronger inhibition of structural damage in AS.

## Abbreviations

2yFU: 2-year follow-up; AS: ankylosing spondylitis; BL: baseline; CI: confidence interval; MRI: magnetic resonance imaging; OR: odds ratio; STIR: short-tau-inversion-recovery; T1-post-Gd: T1-post-gadolinium; TNF: tumour necrosis factor; VE: vertebral edge.

## Competing interests

The authors declare that they have no competing interests.

## Authors' contributions

XB helped to conceive of the idea for the study, prepared the data and performed data analysis, and helped to write the manuscript. JL analysed the data, performed the statistical evaluation, and helped to write the manuscript. MR and JS helped to write the manuscript. JB helped to conceive of the idea for the study and to write the manuscript. All authors read and approved the final manuscript.
